# Active Sampling Device for Determining Pollutants in Surface and Pore Water – the *In Situ* Sampler for Biphasic Water Monitoring

**DOI:** 10.1038/srep21886

**Published:** 2016-02-24

**Authors:** Samuel D. Supowit, Isaac B. Roll, Viet D. Dang, Kevin J. Kroll, Nancy D. Denslow, Rolf U. Halden

**Affiliations:** 1 The Biodesign Institute, Center for Environmental Security and Global Security Initiative, 781 E. Terrace Mall, Arizona State University, Tempe, AZ 85287-5904; 2 Department of Physiological Sciences and Center for Environmental and Human Toxicology, University of Florida, Gainesville, FL 32611.

## Abstract

We designed and evaluated an active sampling device, using as analytical targets a family of pesticides purported to contribute to honeybee colony collapse disorder. Simultaneous sampling of bulk water and pore water was accomplished using a low-flow, multi-channel pump to deliver water to an array of solid-phase extraction cartridges. Analytes were separated using either liquid or gas chromatography, and analysis was performed using tandem mass spectrometry (MS/MS). Achieved recoveries of fipronil and degradates in water spiked to nominal concentrations of 0.1, 1, and 10 ng/L ranged from 77 ± 12 to 110 ± 18%. Method detection limits (MDLs) were as low as 0.040–0.8 ng/L. Extraction and quantitation of total fiproles at a wastewater-receiving wetland yielded concentrations in surface water and pore water ranging from 9.9 ± 4.6 to 18.1 ± 4.6 ng/L and 9.1 ± 3.0 to 12.6 ± 2.1 ng/L, respectively. Detected concentrations were statistically indistinguishable from those determined by conventional, more laborious techniques (*p* > 0.2 for the three most abundant fiproles). Aside from offering time-averaged sampling capabilities for two phases simultaneously with picogram-per-liter MDLs, the novel methodology eliminates the need for water and sediment transport via *in situ* solid phase extraction.

The United States Environmental Protection Agency (USEPA) estimates that approximately 10% of all domestic lakes, rivers, and bays harbor sediments are contaminated by chemicals that threaten aquatic wildlife and human health^1^,^2^. Accurate and efficient environmental sampling is therefore integral for the evaluation of inherent risks associated with environmental contamination. Measured concentrations of environmental contaminants are used in compliance reporting, modeling, and risk assessment for biota and humans^3^,^4^. Some contaminants, such as persistent organic pollutants, pose a long-term threat to ecosystems because they can remain in the environment for decades^5^. This issue is complicated by the fact that many persistent pollutants are hydrophobic, with *n-*octanol-water partitioning coefficient (K_OW_) values on the order of 10^4^ or greater. These hydrophobic organic contaminants (HOCs) are mostly sequestered by organic carbon (OC) in sediments, often irreversibly^6^,^7^. As a consequence, the total sediment concentration may not provide a good representation of the bioaccessible concentration of hydrophobic chemicals, particularly in the quiescent phase inherent to sediment pore spaces^6^,^8^. Aggressive sediment extraction using organic solvents in conjunction with the standard Soxhlet extraction apparatus can facilitate determination of the total sediment contaminant burden. Sediment concentrations then may be used to calculate presumed pore water concentrations, if the system is at equilibrium, by normalizing against the sediment distribution coefficient K_D_ (equation 1)^9^, which is related to sediment organic carbon content:


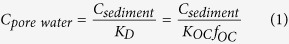


This method of estimation seeks to account for sorption resulting from the organic carbon fraction (f_OC_) in the sediment. The organic carbon partitioning coefficient (K_OC_) can be directly measured, or estimated from the corresponding K_OW_ value^10^. This method of assessing chemical activity in pore water is in wide use for estimating bioavailability, and by extension the ecological risk posed by “truly dissolved” chemicals; however, it does not account for additional, potentially mobile chemical mass associated with colloids and dissolved organic matter^8^,^11^.

Passive sampling is a popular method of *in situ* pre-concentration used for determining chemical activity, frequently as a proxy for assessing bioavailability of sediment-borne pollutants^12^,^13^,^14^. Calibration of passive samplers requires either equilibrium or linear uptake isotherms, often supplemented by the use of performance reference compounds (PRCs) for quality control. The time required by HOCs to reach equilibrium may be on the order of weeks to months, as exemplified by studies using solid phase microextraction (SPME) for the pesticide dichlorodiphenyltrichloroethane or DDT (18 d), or low density polyethylene (LDPE) strips for field sampling of large polycyclic aromatic hydrocarbons (>119 d)^15^,^16^,^17^. Passive samplers are relatively inexpensive, reliable, and well-suited to estimate the chemical activity of truly dissolved compounds^15^,^16^,^17^. In some configurations, they also can enable the determination of time-averaged concentrations^17^.

Active samplers offer an alternate function, in that they can capture the mass of analytes associated with colloidal dissolved organic matter (DOM)^18^ and suspended fine particulates in addition to truly dissolved species. Automatic active sampling offers the benefit of short sampling durations. Deployment times achieved by automatic active samplers may be considerably shorter than those required by passive samplers relying on equilibrium approaches or the use of PRC calibrants. The Continuous Low Level Aquatic Monitoring (C.L.A.M.) device is one such active sampler; it automatically extracts tens of liters of water in one to two days, and utilizes a low-energy-consumption diaphragm pump to pull water through a solid phase extraction disk^19^. It can achieve detection limits in the parts-per-quadrillion range for several hydrophobic organic compounds, by extracting a single composite sample of bulk water per deployment^19^, but it is not designed to sample pore water.

The present work focused on the production of an active sampler that can simultaneously determine bulk water and pore water contaminant levels over long durations to yield time-averaged concentrations of chemical mass in water, whether fully dissolved, or partitioned onto DOM, colloids, and suspended particulates (<30 μm; Supplementary Information, Figure S1). To illustrate the utility of the sampler described herein, we deployed it in an engineered wetland to monitor fipronil and its transformation products (Supplementary Information, Figure S2). These compounds are collectively referred to as fiproles, and have been hypothesized to play a role in the ongoing worldwide honeybee colony collapse disorder^20^,^21^. Fipronil is a halogenated pesticide and emerging contaminant recently banned for most agricultural uses in the European Union^22^. Used in common urban and agricultural applications, it is the active ingredient in many termite treatments, turf treatments, and in agricultural pesticide formulations, commonly in the form of seed treatments. Fiproles are known to occur in urban surface waters, and have been observed in at least one study to exceed aquatic toxicity benchmarks (ranging from 0.011–0.11 μg/L for various fiproles) in over 70% of samples (*n* = 94) from Orange County, CA, for both fipronil and fipronil sulfone^23^. Fipronil also has been quantified in conventional wastewater treatment plants, wherein removal by activated sludge was limited to 18 ± 22%^24^. Fipronil is bioaccumulative (log K_OW_ values for fiproles range from 4.0 to 5.43)^25^,^26^ and toxic to a number of aquatic benthic invertebrates at part-per-trillion (ppt) levels^27^,^28^.

The objectives of this study were to (1) design a device that actively samples both the bulk water and sediment pore water of surface water environments, and (2) to demonstrate its utility for *in situ* pre-concentration of environmentally relevant hydrophobic targets, namely fipronil and four of its immediate degradates, at environmentally relevant concentrations in the parts-per-trillion range. One means of validating the efficacy of the IS2B was to compare the results that it yields with those obtained using conventional sampling methods (e.g., grab sampling). The study further was designed to (3) provide data on a group of emerging contaminants speculated to play a role in the ongoing, worldwide honeybee collapse disorder. The three-part validation study included recovery tests, determination of method detection limits (MDLs), and a quantitative analysis of surface water and pore water using the innovative sampler introduced herein.

## Results and Discussion

### Sampler design, fabrication, and optimization

**Figure 1:**
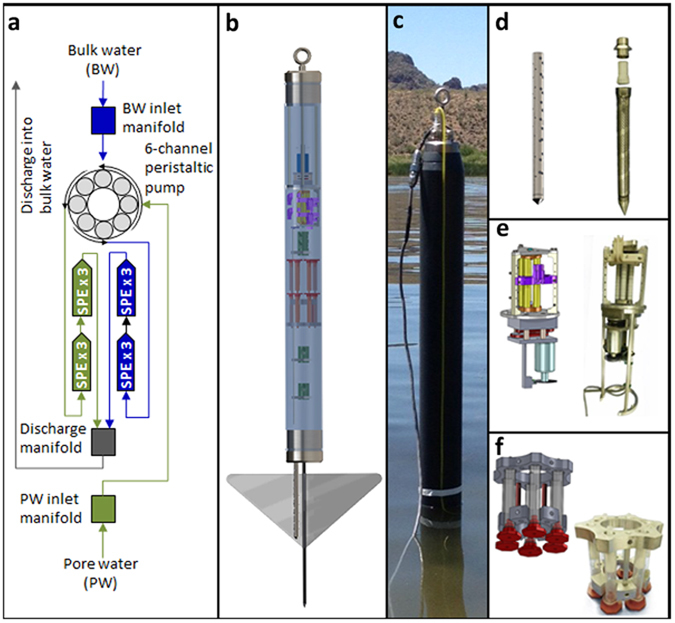
Overview of the IS2B dual-phase sampling methodology and hardware showing: a flow diagram illustrating the extraction process for simultaneous sampling and extraction of bulk and pore water (**a**); computer-aided design drawing of an assembled IS2B unit (**b**); photo of an IS2B deployed in surface water in Arizona, USA (**c**); detailed drawings of (**d**) the sediment pore water inlet spike (right) harboring the perforated inlet screen (left), and (**e**) the pump assembly with mounting frame (right) securing the modified ISMATEC pump (left); also shown are (**f**) the caddy with solid phase extraction cartridges (right) fabricated using the computer-generated blueprint (left).

A functional IS2B sampler was designed in the Center for Environmental Security at Arizona State University (ASU) using SolidWorks® design software, and was fabricated by the ASU machine shop (Fig. 1). The pump delivered water to an array of preconditioned solid phase extraction cartridges, which were connected to the Luer fittings at the pump tubing outlets by adapters (SPE syringe to male Luer slip fitting). The sampler was designed to be embedded in wet sediment, and have discrete inlets both above and below the sediment-water interface. The pore water and bulk water inlets each deliver water to a manifold that divides the flow stream into three streams, and a peristaltic pump delivers each of these streams to an SPE cartridge; there were three cartridges for pore water, and three cartridges for bulk water, all in parallel. An optional second array of cartridges can be included to catch breakthrough (as was done during this study). Cartridges may be selected to match analytes for a given study; in principle, cartridges can be reused but this option was not pursued in this work.

The external parts of the sampler, including the shell, inlets, and fittings are made of stainless steel, and the inlet tubing is made from polytetrafluoroethylene (PTFE). The materials were chosen to minimize chemical interaction with water matrices and analytes. Internal tubing materials chosen were PharMed, PTFE, and Viton for the 2-stop pump cartridges, influent manifold connections, and effluent tubing, respectively. The pore water inlet design includes a perforated steel spike with 1 mm holes to screen out large particulates; the pore water inlet tube within the spike is fitted with a stainless steel mesh. This 2-stage screening system was found to be effective in excluding particulates larger than 30 μm in diameter and producing minimal filter cake on the SPE frits, even after pumping several hundreds of mL of water featuring a high dissolved organic carbon (DOC) content. In this configuration, clogging of the unit was not observed even when it was challenged by placement in fine, high OC soils (f_OC_ = 0.3) with metering pumps set to flow rates of 150 μL/min. More information on the efficacy of the pore water filtration device can be found in the supplementary information online.

### Method performance

The average volume of water delivered (flow rate = 70 μL/min/channel) to a given channel was 203.3 ± 13.9 mL (±6% RSD). Detailed data regarding the volumes delivered can be found in the supplementary information. The relative error of ±6% for the volume delivered to each cartridge was low and acceptable for inferring the precision of subsequent concentration calculations using this sampler.

The method detection limit for fipronil-desulfinyl using IS2B-GC-MS/MS was 0.04 ng/L. The method detection limits for the other four analytes using IS2B-LC-MS/MS ranged from 0.4 to 0.8 ng/L, and are presented along with benchtop analyte recovery rates in Table 1. Average absolute recovery rates in water spiked to 10 ng/L (1 ng/L for fipronil-desulfinyl) were between 82 ± 14% and 110 ± 18%, as determined from 8 replicates. At the lower analyte levels presented in Table 1 (0.1 and 1 ng/L for fipronil-desulfinyl and the other fiproles, respectively), absolute recoveries ranged from 77 ± 12% to 95 ± 13%. Subsequent analyses of SDB-extracted spiked wetland water from this location indicated little to no ion suppression, illustrating that the extraction method is sufficient for reducing matrix effects. These detection limits and performance data are comparable to prior work using off-line extraction and analytical methods for fiproles^29^. One study team used SPE columns to concentrate water samples and analyzed for four fiprole residues (excluding fipronil amide) via gas chromatography tandem mass spectrometry (GC-MS/MS), yielding MDLs from 1.6 to 7.0 ng/L, while absolute recoveries ranged from 73 ± 15 to 110 ± 3%^29^. Thus, the performance of the here presented method compares favorably to previously established, alternative approaches. Details regarding MDL and limit of quantitation (LOQ) calculations can be found in the supplementary information.

### Field study

**Figure 2:**
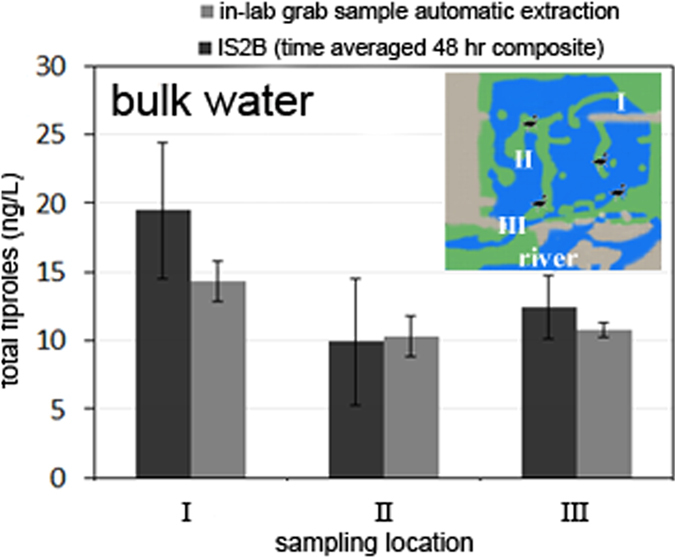
Bulk water concentrations of total fiproles obtained for time-discrete grab samples and for time-averaged, 48-hour composites acquired and extracted *in situ* using the IS2B device. Upper right panel is a schematic of the IS2B field deployment in a constructed wetland in Arizona, USA, showing the flow path of water from sampling locations I (wetland mouth) to II (mid point) to III (outfall into an agricultural irrigation stream). A wastewater treatment plant effluent enters the wetland at location I. The representation of the wetland was drawn in Photoshop by referencing schematics.

Grab samples of water and sediment were taken from three locations in an undisclosed wetland in the southwestern United States (Fig. 2); simultaneously with the IS2B deployment, grab samples were taken at the study location. The wetland in this study is the outfall from a wastewater treatment plant that performs primary through tertiary treatment, with minimal chlorine disinfection; this wetland was designed as a quaternary treatment unit operation. Results are discussed hereafter in the context of the matrix sampled.

### Bulk water

Mean total fiprole concentrations obtained from IS2B sampling of bulk water at the wetland ranged from 9.9–18.1 ng/L. Total fiprole concentrations in bulk water derived from in-lab sample concentration ranged from 10.3–13.4 ng/L. In all but one case (fipronil sulfide at location I), individual bulk water fiprole concentrations derived using these two methods were not discernably different (see Table 2). Individual fiprole concentrations determined using these two methods also were similar. Concentrations of total fiproles in wetland bulk water, as determined using the IS2B, were similar to results from grab sampling coupled with in-lab concentration of analytes from water samples (Fig. 2). Average individual fiprole concentrations, as determined by *in situ* analyte concentration, were statistically indistinguishable from data obtained using a benchtop, large-volume, laboratory extraction apparatus (LEA), or Autotrace 280, for the three most abundant congeners: fipronil (*p* = 0.27), fipronil sulfide (*p* = 0.26), and fipronil sulfone (*p* = 0.22). In addition, *in situ* extraction served to detect fipronil-desulfinyl in all three sampling locations at levels near the detection limit of 0.04 ng/g, whereas no peaks were detected using in-lab LEA processing of grab samples taken from locations I and II. In-lab LEA did yield a desulfinyl peak on the chromatogram obtained for sampling at location III, but it was just below the detection limit of 0.05 ng/L. Fipronil-desulfinyl was detected at location II using the IS2B at 0.35 ± 0.16 ng/L, but was not detected in the grab sample. This could be due to either intermittent presence of the compound (i.e., absence during the discrete grab sampling event relative to the continuous sampling regime intrinsic to the IS2B) or possibly adsorption of fipronil-desulfinyl to the media bottle during storage at 4 °C for a week. The fact that the IS2B allowed this compound to be detected while conventional grab sampling did not highlights the benefits afforded by continuous *in situ* extraction of target compounds: increased likelihood of capturing transient mass fluxes and mitigation of potential sample handling losses.

### Pore water

Mean concentrations of total fiproles in pore water, as determined with the IS2B approach at the three sampling locations, ranged from 9.1–12.6 ng/L, whereas mean total fiprole levels determined using in-lab extraction of pore water from sediment from Site A showed an almost identical value of 8.6 ± 1.4 ng/L. The concentrations of individual fiproles in pore water, as determined using the IS2B, were similar to contaminant levels observed in bulk water (see Table 2). One explanation for this observation could be the occurrence of short-circuiting of liquids from the bulk water to the pore water intake. However, this scenario is not likely when considering that the volume of pore water sampled by the IS2B (600 mL total) is small compared to the theoretical volume of influence around the pore water inlets (1.8 L), and further considering the very slow pump rate of only 70 μL/min. Since short-circuiting of fluids was never observed during laboratory testing, a second, more plausible explanation is that non-equilibrium conditions were extant at the sampling site. Indeed, surface sediments are known to represent dynamic systems that frequently are not at equilibrium with respect to chemical transfer between sediment and pore water^30^,^31^. Probing for such non-equilibrium conditions using equation 3, we determined the organic carbon content of the sediment (1%) using a total organic content analyzer (see supplementary information) and compared the sediment-associated analyte concentration to what was found in pore water. Calculated based on the pore water concentrations measured in sediment processed in the laboratory, the expected sorbed fipronil concentration was estimated at approximately 350 pg/g dry weight sediment, whereas analytical results from solvent extraction of dry sediment yielded a value of less than the MDL or less than 20 pg/g dw. Taken together, these results strongly suggest that non-equilibrium conditions prevailed between the sediment and pore water in this highly dynamic sampling location downstream of an urban wastewater treatment plant that is known to experience notable seasonal and diurnal fluctuations in both flow rates and concentrations of water constituents. Our findings are consistent with prior studies by other groups showing that predictive models for estimating sediment concentrations of non-ionic pesticides and other HOCs from aqueous concentrations may overestimate sorbed fractions^30^,^31^,^32^,^33^. Overall, modeling of the fate of pesticides is known to be challenging as environmental factors extant at field sites, such as the one studied here, may not be consistent with the assumptions required to employ equilibrium models^32^.

### Toxicological implications of monitoring results

Aqueous concentrations of fiproles determined here for a constructed wetland in the southwestern U.S. are lower than those reported previously for urban settings but within one order of magnitude of levels known to be toxic to aquatic biota. Various aquatic organisms are highly susceptible to fipronil, as illustrated by the data compiled in Table 3. Concentrations detected in this study were below the toxic threshold values of reference organisms, and also lower than those detected in urban streams in California^23^,^34^,^35^. In one study where fiproles were quantified in urban runoff in Orange County, median combined fiprole concentrations ranged from 204–440 ng/L^34^. Possibly influenced by different land use patterns, these values are an order of magnitude higher than the mean bulk water concentrations determined in this study (10–18 ng/L). Another 6-month monitoring study of the Rhône River in France in 2004 reported no detections of fipronil with a limit of detection of 1 ng/L, a finding that is consistent with the European ban of fipronil application in agriculture^36^. However, fipronil remains in widespread use in the United States, which explains the detections reported here and by the few additional data available for America^23^,^34^,^35^.

### Technology applicability

The IS2B technology is intended as a means for concentrating trace level chemicals *in situ*, which eliminates the need to transport the large volumes of water needed for in-lab analysis. Whereas the volume of water assayed in this study was 1.2 L in total, sampling of larger or smaller volumes can easily be accomplished, with the selection of the water volume being a function of contaminant concentration, method detection limits, etc. When seeking to process very large volumes of pore water, maintaining equilibrium conditions may impose flow rate limits. Since the pore water inlet is about 15 cm below the sediment-bulk-water interface, a spherical volume of 1.8 L of pore water 
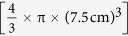
 represents the upper limit of sediment pore water volume processed. In contrast to pore water, the volume of surface water that can be sampled is limited only by the capacity of the resin cartridges used, and can be tens, or even hundreds of liters. In this study, cartridges were deployed in series for one of the channel replicates to verify that breakthrough was not occurring. This low-flow, dual-phase sampling capability is the significant advantage of the IS2B over devices like the C.L.A.M., which is designed to sample large volumes of only bulk water very quickly. The pump on the C.L.A.M. uses very little energy, but it is not capable of the sub-mL/min flow rates of the peristaltic pump installed on the IS2B; nor is it capable of biphasic sampling. The design of the IS2B allows for the pumping of pore water to be slow enough to avoid rapid intrusion of bulk water from above the sediment-water interface.

As performed here, taking of samples with three replicates of each phase (bulk water and pore water) is the preferred deployment mode. This redundancy enables the determination of standard deviations and guarantees the availability of useful data in the event of isolated technical problems, such spillage of extracts during handling in the laboratory. As demonstrated here for fiproles, tubing material should be matched to the chemistry of the analyte to avoid losses from sorption.

### Conclusions

The IS2B technology is complementary to established passive sampling and grab sampling strategies by offering several attractive attributes: (i) determination of time-averaged concentrations in triplicate in a single deployment; (ii) concentration measurements for two distinct environmental phases simultaneously (bulk and pore water); (iii) obtained contaminant levels are reflective of the total quantity of mobile, potentially bioaccessible contaminants in surface water and sediments^8^, concentrations which may differ substantially from the chemical activity of truly dissolved solutes (which are best determined using established passive sampling strategies); (iv) the IS2B concentrates analytes on SPE resins in the field, which eliminates the need to collect large volumes of time-discrete water samples via either grab sampling by hand or by using other automated water collectors; (v) avoidance of the need to transport several kilograms of wet sediment to the lab for determination of pore water concentrations; (vi) sample handling steps and opportunities for lab contamination are reduced due to *in situ* analyte concentration; (vii) shipping costs are reduced since the analyte-laden resin cartridge weighs only a fraction of the large mass of water that was extracted in the field; and (viii) the technology is well-suited for measurements in highly dynamic environmental compartments where non-equilibrium conditions are expected to prevail. Additionally, the IS2B offers advantages over other manual or automated active sampling technologies: Rhizon samplers^37^ and MINIPOINT^38^ samplers are both designed to obtain time-discrete samples of only pore water, whereas the IS2B is designed to obtain time-averaged samples of both pore water and bulk water; automated water samplers like ISCO®^39^ or Hach® Sigma^40^ samplers are designed to collect bulk water at relatively high flow rates and store it in bottles, whereas the IS2B can collect both bulk and pore water simultaneously and separately, and can do so at low flow rates while also extracting target analytes *in situ*, which minimizes sample handling losses, reduces the risk of contamination, and may improve the stability of analytes due to capture by the adsorbing resin.

## Methods

### Sampler design

Design drawings were produced using Solid Works® design software (Dassault Systèmes SOLIDWORKS Corp., Waltham, MA). Active sampling by the *in situ* sampler for biphasic water monitoring (IS2B) device was facilitated by a low-flow, multi-channel peristaltic pump capable of pumping at a continuous rate of 70 μL·min^−1^ or less in each of its six channels. The pump head and motor originated from an ISMATEC Reglo-E Digital 12DC, geared at a ratio of 25:1 (IDEX corp., Oak Harbor, WA). The pump was mounted onto an aluminum frame, fit with custom tubing cartridges for compressing the pump tubing. The pump cartridges were designed to fit on the custom frame, and are identical to those used in another environmental monitoring and assessment tools, the *in situ* microcosm array (ISMA)^41^. The pump tubing (0.38 or 0.51 mm inner diameter, ID) consisted of 2-stop PharMed tubing (Saint-Gobain Performance Plastics, Akron, OH), while influent tubing was 6.4 mm ID polytetrafluoroethylene PTFE for the pore water inlet and 1.6 mm PTFE for the bulk water inlet. Effluent tubing is 0.89 mm Viton coupled to 1.6 mm PTFE via a 6-channel manifold. Polyvinylidene fluoride (PVDF) Luer fittings and all tubing were purchased from Cole Parmer (Vernon Hills, IL). SPE to Luer slip adaptor fittings were purchased from Sigma Aldrich (St. Louis, MO). All SPE cartridges were purchased from Phenomenex (Torrance, CA). Polystyrene divinylbenzene resin (Strata SDB-L) was chosen for its affinity for hydrophobic, aromatic pesticides like fipronil. The pore water inlet was incorporated into a perforated stainless steel (SS) tube with 1 mm holes, and the tube itself was wrapped in several layers of a SS mesh screen with 30 μm openings. Metal for the inlet apparatus was purchased from Grainger (Lake Forest, IL), and the ASU machine shop fashioned it into a 20-cm inlet spike that could be driven into wet sediment. The unit was contained within an 8.9-cm outer diameter (OD) stainless steel tube, capped at each end by threaded caps with compression fittings for wiring and tubing. Compression fittings were purchased from McMaster-Carr (Santa Fe Springs, CA). A stainless steel Swagelok® adaptor for outlet tubing compression was purchased from Swagelok Company (Solon, OH). The peristaltic pump motor was powered by a 12-V Optima Blue Top battery with a power inverter, and controlled by an external ISMATEC MiniClick6 Reglo-E control unit, purchased from IDEX. The 9455 multi-conductor control and instrumentation cables were purchased from Belden (St. Louis, MO). In deployment configuration, the battery, power inverter, cable spool, and control unit were stored in a deck box onshore.

### Instruments and analysis

All analytes aside from fipronil-desulfinyl were quantified by performing multiple reaction monitoring (MRM) using liquid chromatography electrospray tandem mass spectrometry (LC-ESI-MS/MS) operating in negative mode. For enhanced method sensitivity, fipronil-desulfinyl was analyzed by MRM using gas chromatography electron ionization tandem mass spectrometry (GC-MS/MS). Details about instrument parameters for methods developed specifically for this study can be found in the supplementary information.

### Sediment collection and analysis

Prior to the case study, approximately 500 g of wet sediment was collected from a wetland at the specific locations where the sampler was deployed. Triplicate aliquots of wet sediment weighing about 1 g each were dried under a nitrogen stream, and subsequently weighed to the nearest milligram. The sediment samples were then extracted with 2 mL of 1:1 hexane/acetone (v/v) in a sonicator for 3 h. The extracts were blown down to dryness and reconstituted to 2 mL of acetonitrile and sonicated for 20 minutes. The resulting samples were filtered with 0.2 μm PTFE filters before analysis via liquid chromatography with tandem mass spectrometry (LC-MS/MS). LC samples were diluted by 50% with LC-MS grade water prior to sample injection.

### Benchtop water collection and analysis

Two hundred milliliters of bulk surface water was collected from each deployment site in ashed amber media bottles at the time of IS2B deployment and stored at 4 °C for about a week. Water samples were extracted by an automatic large volume extraction apparatus (Autotrace 280), using a similar SPE and analysis method as described for the IS2B (see supplementary information). In addition, approximately 10 L of wet sediment was collected from site A, placed into a 19-liter bucket, and stored at 4 °C. In order to extract pore water from the sediment grab sample in the lab, the perforated IS2B inlet spike was assembled, and three lines from a 6-channel automated SPE unit were wrapped in 30 μm stainless steel mesh and secured with PTFE tape before being placed inside the perforated tube. The inlet spike was thrust into the sediment far enough to ensure an inlet depth of at least 8 cm. The water was then automatically extracted in triplicate alongside Milli-Q water unspiked controls in duplicate using a large volume automatic solid phase extraction unit, Cartridges were conditioned with 3 mL of acetonitrile, and equilibrated with 3 mL of Milli-Q water. They were then loaded with 200 mL of water, and eluted serially with 2 mL acetonitrile, followed by 2 mL of hexane:acetone (1:1). Serial eluates for each cartridge were combined, and two 0.5 mL aliquots were taken from each sample to be evaporated under nitrogen. One aliquot from each sample was reconstituted to 0.5 mL acetonitrile and diluted to 1.0 mL with water for analysis by LC-MS/MS. The other aliquot of each sample was reconstituted to 0.5 mL hexane for analysis by GC-MS/MS. Overlaying water from the same locations was collected in oven-cleaned 1 L media bottles (~1 L per bottle was collected) and stored at 4 °C before being extracted as described above. A field blank, consisting of ultrapure reagent grade water transferred to an oven-cleaned bottle onsite, was also extracted, and the resultant signal from the field blank chromatogram was subtracted from those obtained for the bulk water and pore water extracts. For all analytes, field blank chromatograms showed no to <10% of the signal intensity obtained in chromatograms for actual samples.

### Calibration

The IS2B peristaltic pump can be programmed to accommodate various pump tubing diameters; after inputting the tubing diameter and calibrating at a given flow rate, the control unit can be reprogrammed for a different flow rate while maintaining its calibration. Performance was verified by pumping and measuring 1 mL aliquots at a flow rate half that of the calibrated flow rate.

Prior to all tests, the peristaltic pump was rinsed with approximately 150 mL of denatured ethanol and then with an equal volume of 18.2 MΩ water to prime the tubing. The control unit was set to deliver 10 or 20 mL to each of the six channels, and the tubing cartridges were then adjusted to even the flow rates to each channel. Once calibrated, the pump was set to deliver 200 mL of water to each channel at a flow rate of 140 μL/min, and the effluent was captured in pre-weighed 250 mL media bottles.

### Field study

The fully assembled IS2B was calibrated for a flow rate of 70 μL/min/channel and deployed in a wetlands receiving runoff from a wastewater treatment facility, and from adjacent agricultural fields. The flow rate was chosen in order to minimize the chance for bulk water penetration into the pore spaces as a result of drawdown. A validation study for another pore water sampling device called MINIPOINT indicated that for a sediment with an average porosity of 0.35, flow rates lower than 4000 μL/min did not disturb tracer (Cl^−^) depth profiles, and the lowest flow rate evaluated was 300 μL/min^42^. The total flow rate into the IS2B from the pore spaces in this field deployment was 70 μL/min/channel × 3 channels, or 210 μL/min, which is lower than the lowest flow rate validated for the MINIPOINT. At each deployment location the IS2B simultaneously drew pore water and overlaying bulk water through separate inlets and delivered 200 mL to each of 6 new, conditioned 25 mg/1 mL SDB cartridges arranged in parallel at 70 μL/min/channel. A second array of 6 of C-18 cartridges (100 mg/1 mL) were placed behind the SDB cartridges in series to assess breakthrough of fiproles. The extracted water was released into the bulk water phase, downstream of the bulk water inlet. The SPE cartridges were extracted and the samples processed as described above.

Sediment and water samples were collected concurrent with sampler deployment. One-gram aliquots of sediment were dried, weighed, spiked with 5 ng fipronil des F3 (C_12_H_7_Cl_2_F3N_4_OS) as a surrogate, and extracted with 2 mL hexane:acetone for 30 min in a sonicator. One mL of the supernatant was drawn and evaporated under a nitrogen stream. After solvent exchange into 100% acetonitrile, extracts were filtered with 0.2 μm PTFE, and 500 μL of the filtrate was combined with 500 μL LC-MS grade water for injection into a high-performance liquid chromatography-tandem mass spectrometer (HPLC-MS/MS). Sediment concentration was calculated using equation 2:


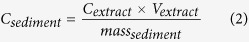


The pore water concentration (*C*_*PW*_) then was inferred by normalizing the sediment concentration by the distribution coefficient (*K*_*D*_) as described in equation 3:


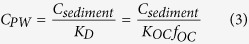


*K*_*OC*_ was estimated using the published linear relationship shown in equation 4^10^,^43^,^44^,^45^


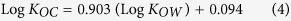


The fraction of organic carbon in sediment (*f*_*OC*_) was determined by total organic carbon (TOC) analysis as described in the supplementary information.

### Statistical data analysis

Comparison of the mean bulk water concentrations as determined by IS2B sampling and grab sampling was done by performing two-tailed *t*-tests at the 95% confidence interval, assuming equal variances. The mean bulk water concentration (log-transformed) of each of the three most abundant congeners (fipronil, and the sulfide and sulfone degradates) was calculated separately for each analyte concentration method (IS2B or LEA), using data from all three sampling locations (*n* = 12), and each sample was assessed for normal distribution. Fipronil amide and fipronil-desulfinyl were omitted from the mean comparison analysis because most peak areas were near or below the method detection limit, thereby producing non-normal distributions. Statistical calculations were performed using the Microsoft Office 2010 Data Analysis ToolPak.

## Additional Information

**How to cite this article**: Supowit, S. D. *et al.* Active Sampling Device for Determining Pollutants in Surface and Pore Water - the *In Situ* Sampler for Biphasic Water Monitoring. *Sci. Rep.* **6**, 21886; doi: 10.1038/srep21886 (2016).

## Supplementary Material

Supplementary Information

## Figures and Tables

**Table 1:** Calculated method detection limits (MDLs) and limits of quantitation (LOQs) for fiprole congeners for using either a conventional large-volume laboratory extraction apparatus (LEA) for pre-concentration or the IS2B technology (*n* = 7).

	Chemical	MDL (ng/L)	LOQ (ng/L)	Recovery (%)	RSD (%)	Spike (ng/L)
LEA	Fipronil	0.9	3	72	27	1
	-sulfide	0.7	2	87	23	1
	-sulfone	1.0	3	87	31	1
	-amide	0.8	3	93	25	1
	-desulfinyl	0.05	0.2	74	15	0.1
IS2B	Fipronil	0.7	2	92	24	1
	-sulfide	0.7	2	93	22	1
	-sulfone	0.4	1	86	9	1
	-amide	0.8	3	77	12	1
	-desulfinyl	0.04	0.1	95	13	0.1

**Table 2:** Concentrations of fiproles in ng/L as determined by *in situ* pre-concentration of samples using the IS2B device, and by grab sampling and extraction of large-volume samples using an automated extraction apparatus in the laboratory.

Chemical			Fipronil	-Sulfide	-Sulfone	-Amide	-Desulfinyl	Total fiproles
I	BW	IS2B	14.1±3.3	ND (<0.7)	4.0±1.3	ND (<0.8)	0.04±0.14^*a*^	18.1±4.6
		LEA	10.0±0.8	ND (<0.7)	3.4±0.5	ND (<0.8)	ND (<0.05)	13.4±1.3
	PW	IS2B**	7.5±1.0	1.4±0.4^a^	3.7±0.7	ND (<0.8)	ND (<0.04)	12.6±2.1
		LEA	5.3±0.2	1.4±0.5^a^	1.9±0.7	ND (<0.8)	ND (<0.05)	8.6±1.4
II	BW	IS2B	5.0±2.5	0.8±0.5^a^	2.3±0.9	1.4±0.7^a^	0.35±0.16^a^	9.9±4.6
		LEA	3.0±0.1	2.8±1.1	2.2±0.1	2.3±0.2^a^	ND (<0.05)	10.3±1.5
	PW	IS2B*	5.6	0.94^a^	2.9	2.0^*a*^	0.3	11.6
III	BW	IS2B	5.4±0.8	0.8±0.1^a^	3.7±0.9	2.4±0.4^a^	0.06±0.11^a^	12.4±2.3
		LEA	4.6±0.2	0.8±0.1^a^	3.3±0.1	2.0±0.1^a^	ND (<0.05)	10.7±0.5
	PW	IS2B	4.2±1.4	ND (<0.7)	2.9±1.0	1.9±0.5^a^	0.09±0.08^a^	9.1±3.0

Sampling locations I, II, and III are those referenced in Fig. 2.

BW, bulk water; PW, pore water; LEA, laboratory extraction apparatus (large volume).

Standard deviations shown are calculated from *n* = 3, except where indicated.

^a^values are below the limit of quantitation, and are therefore estimated.

^*^n = 1 field replicate (2-day, time-averaged composite).

^**^n = 2 field replicates (2-day, time-averaged composite; ±values provided represent maximum/minimum).

**Table 3:** Data on toxicity, occurrence, and persistence of fipronil and three of its degradates.

Compound	Formula	*Procambarus*^a^	*Hyalella azteca*^b^		*Diphetor hageni*^b^			Half-life	
		^43^LC_50_ (μg/L)	^28^LC_50_ (μg/L)	^28^EC_50_ (μg/L)	^28^LC_50_ (μg/L)	^28^EC_50_ (μg/L)	^42^OC urban water conc. (μg/L)	^44^Silt loam (days)	^45^Facultative conditions (days)
Fipronil	C_12_H_4_Cl_2_F_6_N_4_OS	14.3–19.5	1.3–2.0	0.65–0.83	0.20–0.57	0.11–0.21	0.05–0.39	21±0.15	–
-desulfinyl	C_12_H_4_Cl_2_F_6_N_4_	68.6	–	–	–	–	0.05-0.13	–	217–497
-sulfide	C_12_H_4_Cl_2_F_6_N_4_S	15.5	1.1–1.7	0.007–0.003	–	–	ND	>200	195–352
-sulfone	C_12_H_4_Cl_2_F_6_N_4_SO_2_	11.2	0.35–0.92	0.12–0.31	0.19–0.54	0.055–0.13	0.05–0.19	>200	502–589

OC, Orange County, California.

ND, not detected.

^a^*Procambarus* species were *clarkii* and *zonangulus*.

^b^Values for *H. azteca* and *D. hageni* represent the 95% confidence interval.
