# Supplementary material for: Active Sampling Device for Determining Pollutants in Surface and Pore Water – the In Situ Sampler for Biphasic Water Monitoring
Source: Sci Rep. 2016 Feb 24;6:21886. doi: 10.1038/srep21886 (PMC4764808; doi:10.1038/srep21886)
Supplement: Supplementary Information [file srep21886-s1.pdf]

Supplementary Information

Active Sampling Device for Determining Pollutants in Surface and Pore Water – the In Situ Sampler for Biphasic Water Monitoring

Samuel D. Supowit, Isaac B. Roll, Viet D. Dang, Kevin J. Kroll, Nancy D. Denslow, Rolf U. Halden*

* Rolf U. Halden; (P): 480-727-0893; email: Rolf.Halden@asu.edu

Pages (13)

Figures (2)

Tables (2)

Quality Assurance

Solvents and standards. Neat analytical standards of fipronil and fipronil-desulfinyl were purchased from Sigma Aldrich (St. Louis, MO). Neat analytical standards of fipronil sulfide, sulfone, and amide were manufactured by Bayer and Basf (Ludwigshafen, Germany). Organic solvents and Fluka brand liquid chromatography mass spectrometry (LCMS) grade water were purchased from Sigma Aldrich, while Optima brand LCMS grade water was purchased from Fisher Scientific (Fair Lawn, NJ). Fipronil des F3 (a fipronil analog) was purchased from Dr. Ehrenstorfer labs (Augsburg, Germany). Individual standard solutions of the target compounds were prepared by dissolving 10 mg neat standard into 10.0 mL of acetonitrile, or toluene, in the case of fipronil desulfinyl. Solutions were then vortexed until dissolution was complete, yielding 1.0 g/L standards from which serial dilution produced commingled standard solutions ranging from 5 mg/L to 1 µg/L in acetonitrile. Separate standards of fipronil desulfinyl were prepared for GC-MS/MS calibration in hexane.

Pump performance. The IS2B peristaltic pump was calibrated prior to each analysis. Two replicate benchtop tests were performed to ascertain the precision of the pump. Results are shown in supplementary table S1. Multiple trials with this device indicated that one type of tubing (PharMed) provided greater consistency in pump performance than did others (e.g., Viton), probably due to the tendency of the latter to deform permanently when pinched by the pump rollers. During the recovery tests, the ISMATEC control unit was set to deliver 200 mL at a pump rate of 140 μL/min/channel to each of the six channels in two consecutive runs (n = 12).

Sample collection. Sediment field blanks were collected from locations about 50 yards from the edge of the wetland. The sediment was not impacted by the wastewater effluent, and was

therefore used as a quality control. Water field blanks were DI water samples transported from Arizona State University to the wetland, and transferred there into ashed media bottles.

Analytics. Calibration standard response accuracy had to be within 20% of expected values. Level 1 QA/QC for quantitation of fiproles was performed using lab control spikes. The absolute recovery of spiked mass was compared to "clean" calibration standards in 1:1 acetonitrile:water (for LC-MS/MS analysis) or 100% hexane (for GC-MS/MS analysis), and these results are displayed in supplementary table S2. Unspiked equipment blanks were used as controls, and the method of quantitation required subtraction of the equipment blank signal from that of the spiked samples.

Recovery tests. Water laden with dissolved organic carbon (DOC) was generated by adding 100 mg potassium citrate to 3 L of 18.2 MΩ (Milli-Q) water. The water was spiked to 300 ppm (v/v) with Kathon CG/ICP biocide and stored at room temperature in ashed amber media bottles. 2000 mL was transferred to a 2-L ashed media bottle and was spiked with 20 ng (nominal concentration 10 ng/L) of the fipronil parent compound, along with the sulfide, sulfone, and amide degradates. A separate 2-L sample of water was spiked with 2 ng (nominal concentration 1 ng/L) with fipronil-desulfinyl. Both samples were extracted in separate tests as described below.

For bench top extraction, the sampler was assembled with two 3-channel PTFE manifolds for water inlet, and six 1-mL SDB-L SPE cartridges (25 mg of resin), conditioned and rinsed with acetonitrile and LCMS grade water, respectively. Both IS2B inlet tubes were placed into the spiked lab-created water with the IS2B control unit set to deliver 200 mL at 140 µL/min/channel. The effluent tubes from the SPE cartridge were each placed into separate weighed 1000 mL

media bottles. At the end of the pumping period, the SPE cartridges were rinsed with 1 mL LCMS water, and eluted with 1 mL of acetonitrile, followed by 1 mL of 1:1 hexane:acetone. The serial eluates from each channel were combined, divided into two 1 mL aliquots, evaporated under nitrogen, and one set of aliquots was reconstituted to 1 mL of acetonitrile (ACN), while the other was reconstituted to 1 mL hexane. The resulting ACN solutions were diluted by 50% with water, and the ACN/H2O samples were analyzed by LC-MS/MS for fipronil and the sulfide, sulfone, and amide degradate, while the samples in hexane were analyzed by GC-MS/MS for the desulfinyl degradate. In order to determine the background concentrations of the five analytes in the matrix, 200 mL of lab-created unspiked DOC-laden water was extracted by the IS2B in triplicate, along with 200 mL of 18.2 MΩ water (in triplicate). Absolute recoveries were calculated by the background subtraction method. Reagent blanks returned no peaks, but unspiked control matrices returned peaks with areas similar to the lowest calibration point (10 ng/L).

After the recovery test, the pump calibration was assessed by comparing the set volume on the control unit with the volumes collected in the effluent capture bottles. The volumes were determined by dividing the mass difference between the empty and full bottles by the density of water.

A similar procedure was used to determine the recovery efficiency using an AutoTrace 280 by Dionex (Sunnyvale, CA). Because the AutoTrace was equipped with 3 mL rather thatn 1 mL adaptors, it was loaded with 500 mg/3mL SDB cartridges (8 replicates total), which were conditioned as described above. Benchtop comparisons indicated that the 25 mg and 500 mg resin beds performed similarly, but 500 mg cartridges were on hand for this study. 200 mL of spiked DOC-laden water with 1 ng/L of targets was loaded onto each cartridge at 1 mL/min, and

eluted serially with 2 mL of acetonitrile and 2 mL of hexane:acetone (1:1) at 1 mL/min. The eluates were commingled and blown down to dryness under nitrogen before being reconstituted to 2 mL of acetonitrile. These samples were split for GC-MS/MS analysis and LC-MS/MS analysis. LC samples were diluted by 50% with LCMS grade water prior to analysis. GC samples were solvent switched to hexane prior to analysis.

Method Detection Limit. A sample of lab-generated water (as described above) was used to determine the baseline signal for each analyte. Nine replicate samples were generated, and two were subsequently omitted, resulting in six degrees of freedom. The method detection limit (MDL) was calculated as described by the Environmental Protection Agency.[1] This method was used to determine the MDL using both the AutoTrace and IS2B preconcentration devices. Since the IS2B and AutoTrace each have six channels, the process was run twice: once with three spiked replicates and three unspiked controls, and once with six spiked replicates. A student's t-value (99% confidence interval) of 3.14 was used, and was multiplied by the standard deviation of 7 replicates. The calculated MDLs were checked against the following criteria:

MDL < spike level

Spike level < 10 x MDL

70 % < Absolute recovery < 130%

Signal-to-noise ratio < 10

Porewater filtration. One concern about sampling porewater *in situ* was that mobile particulates would clog the frits of SPE cartridges and inhibit flow. This concern was addressed by (1)

visually inspecting the quality of the filtered porewater (supplementary figure S1), and (2) by measuring volumes of filtered porewater delivered to cartridges.

Sample preparation/breakthrough test

IS2B. The IS2B was set to deliver 200 mL at 70 µL/min and 140 µL/min to 25 mg polystyrene divinylbenzene (SDB) cartridges. In both a lab and field test, 100 mg C18 cartridges were placed downstream of the SDB in order to ascertain whether any analyte mass passed through the initial SDB cartridges. The cartridges were immediately eluted serially with 1 mL acetonitrile and 1 mL 1:1 hexane:acetone. The breakthrough cartridge eluates indicated no fiproles broke through the initial SDB cartridges.

In-lab water sample extractions. The AutoTrace 280 was equipped with 500 mg/3 mL SDB cartridges. The AutoTrace program is as follows:

1. Condition cartridge with 4.0 mL of acetonitrile into aqueous waste.
2. Condition cartridge with 2.0 mL Milli-Q water.
3. Load 200.0 mL of sample onto cartridge.
4. Rinse cartridge with 2.0 mL of Milli-Q water into aqueous waste.
5. Dry cartridge with nitrogen gas for 10.0 minutes.
6. Soak and collect 0.5 mL fraction using acetonitrile.
7. Collect 2.0 mL fraction into sample tube using acetonitrile.
8. Collect 2.0 mL fraction into sample tube using 1:1 hexane:acetone.

All eluates were solvent switched to either 1:1 acetonitrile:water or 100% hexane for LC-MS/MS or GC-MS/MS analysis, respectively.

Instruments and analysis

TOC of sediment samples was analyzed using a Shimadzu TOC Solid Sample Module SSM-5000A (Shimadzu Scientific Instruments, Inc., Columbia, MD), while TOC of water samples was assessed using a Shimadzu TOC-5000 analyzer. Fipronil and the sulfide, sulfone, and amide degradates were quantified using liquid chromatography negative electrospray ionization tandem mass spectrometry (LC-ESI-MS/MS) with background signal subtraction. Fipronil-desulfinyl was quantified using gas chromatography tandem mass spectrometry (GC-MS/MS) with background signal subtraction. LC mass spectrometric analyses were performed using an API-4000 MS/MS (Applied Biosystems, Framingham, MA) coupled to a Shimadzu Prominence HPLC controlled by Analyst 1.5 software (Applied Biosystems, Framingham, MA). Separation was done using an Ultra IBD column (5 µm particle size, 2.1 × 150 mm; Restek Corporation, Bellefonte, PA). The mobile phase consisted of 40% acetonitrile and 60% water flowing at a rate of 400 µL/min with a total runtime of 12 min, with a gradient profile of 10% ACN/min starting at t = 1.00 min. Analytes were introduced into the mass spectrometer using an electrospray ionization probe operating in negative mode, and multiple reaction monitoring (MRM) was used for qualitative analysis. Optimized conditions for the ionization and fragmentation of the analytes are specified below. Quantitation was performed using a 5 point calibration curve in 1:1 acetonitrile:water. GC mass spectrometric analysis was performed using an Agilent 7890 gas chromatograph coupled to an Agilent 7000 triple quadrupole mass spectrometer (Agilent Technologies, Santa Clara, CA) operating in positive mode, and MRM was used for qualitative analysis. Absolute recovery of all compounds was performed by using 4- or 5-point calibration curves and subtracting the concentration in the unspiked matrices from those of the spiked matrices. Equipment blanks using 18.2 MΩ (Milli-Q) water were run prior to all deployments, and grab sample controls included field blanks of Milli-Q water.

GC-MS/MS Method (Agilent 7890 GC/7000 QQQ)

GC parameters

Inj. Volume: 1.0 uL

Oven

70 °C for 0.1 min

then 40 °C/min to 250 °C for 0 min

then 40 °C/min to 300 °C for 0.2 min

5 min (Post Run): 300 °C

Mode: Splitless

Heater: 280 °C

Flow: 1.2 mL/min

Pressure: 10.966 psi

Column: HP-5MS 5% Phenyl Methyl Silox

30 m x 250 μm x 0.25 μm

Mass spectrometer parameters

Fipronil-desulfinyl

Q1 Mass (Da)	Q3 Mass (Da)	Dwell(msec)	CE
388	333	150	25.00

Q1 Mass (Da)	Q3 Mass (Da)	Dwell(msec)	CE
388	281	150	30.00

LC-MS/MS Method

LC parameters (Shimadzu Prominence HPLC)

Shimadzu LC system Injection Volume = 20.00 μl

Pump Model: LC-20AD

Pumping Mode: Binary Flow

Total Flow: 0.4000 mL/min

Time Program

Time (min)	% Methanol
0.01	40
1.00	40
6.00	90
8.00	90
8.50	40
9.00	40
11.00	Stop

Mass spec parameters (AB Sciex API 4000)

Fipronil

Q1 Mass (Da)	Q3 Mass (Da)	Dwell(msec)	DP	CE
434.95	329.90	80.00	-70.00	-24.00

Q1 Mass (Da)	Q3 Mass (Da)	Dwell(msec)	DP	CE
434.95	250.00	80.00	-70.00	-38.00

Fipronil sulfide

Q1 Mass (Da)	Q3 Mass (Da)	Dwell(msec)	DP	CE
418.70	382.80	80.00	-75.00	-18.00

Q1 Mass (Da)	Q3 Mass (Da)	Dwell(msec)	DP	CE
418.70	262.10	80.00	-75.00	-40.00

Fipronil sulfone

Q1 Mass (Da)	Q3 Mass (Da)	Dwell(msec)	DP	CE
450.85	414.71	80.00	-70.00	-40.00

Q1 Mass (Da)	Q3 Mass (Da)	Dwell(msec)	DP	CE
450.85	281.84	80.00	-70.00	-40.00

Fipronil amide

Q1 Mass (Da)	Q3 Mass (Da)	Dwell(msec)	DP	CE
386.74	350.84	80.00	-70.00	-40.00

Q1 Mass (Da)	Q3 Mass (Da)	Dwell(msec)	DP	CE
386.74	281.89	80.00	-70.00	-40.00

Supplementary Table S1. In two replicate runs, each of six channels was calibrated to deliver 200 mL at 140 µL/min. Individual channel volumes were measured by mass, assuming a fluid density of 1.0 g/mL.

Channel	Vol delivered (mL)	Abs error (mL)	*%Error*
Trial 1			
1	203.7	3.7	2%
2	190.8	-9.2	5%
3	202.8	2.8	1%
4	219.7	19.7	10%
5	195.6	-4.4	2%
6	180.8	-19.2	10%
Trial 2			
1	219.1	19.1	10%
2	187.1	-12.9	6%
3	216.5	16.5	8%
4	219.6	19.6	10%
5	192.9	-7.1	4%
6	211.0	11.0	6%
Avg	203.3	3.3	6%
Stdv	13.9		3%

Supplementary Table S2. IS2B absolute recoveries of fiproles from lab water (n = 8).

**Chemical**	**Spike level (ng/L)**	**Recovery (%)**	**SD (%)**
Fipronil	10	103	15
-sulfide	10	82	14
- sulfone	10	89	13
-amide	10	90	14
-desulfinyl	1	110	18

Matrix:

33 mg/L potassium citrate

300 ppm Kathon

Milli-Q water

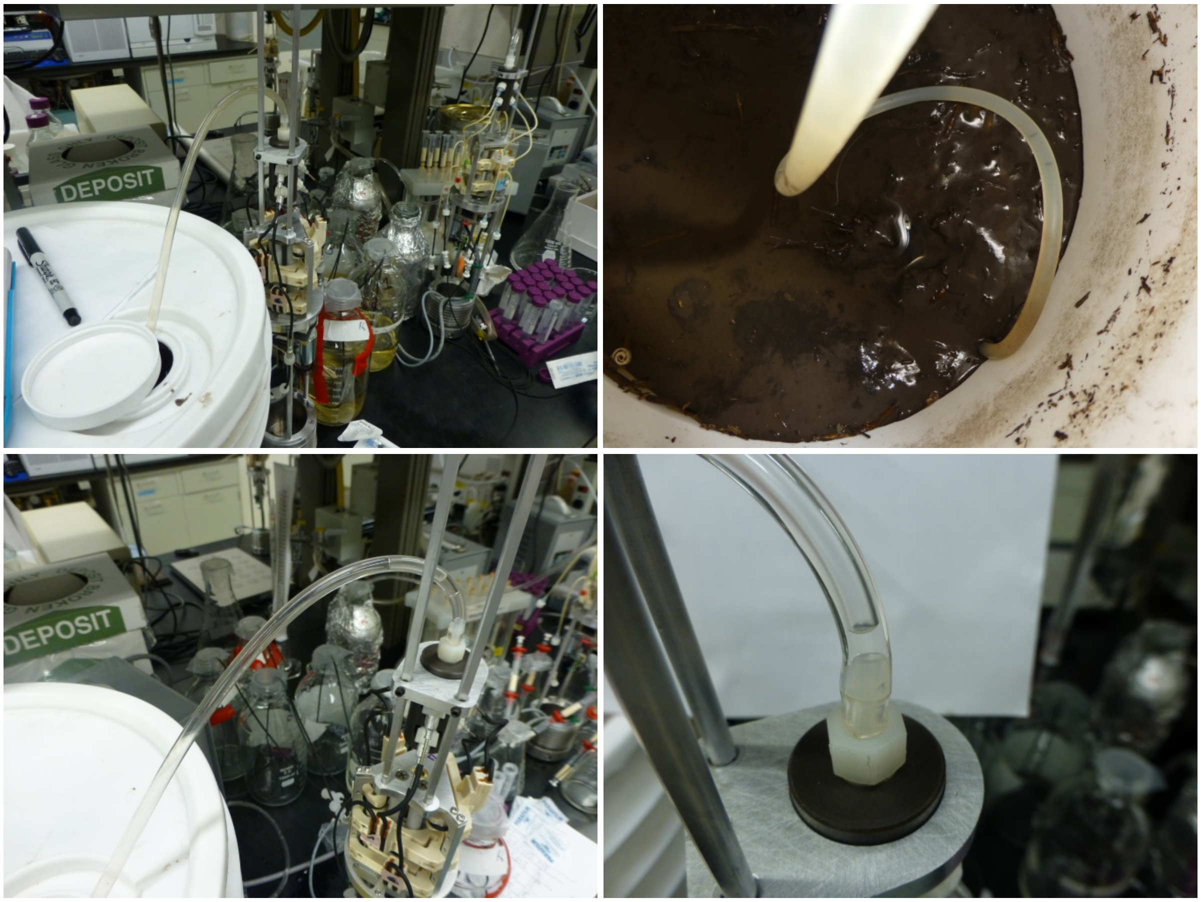

Supplementary Figure S1. The IS2B inlet is immersed in high-OC sediment (> 30% OC) and is drawing water at 100 µL/min. The clear plastic tubing shown carries the filtered pore water. Plastic tubing shown is for demonstration purposes only. Actual inlet tubing is PTFE.

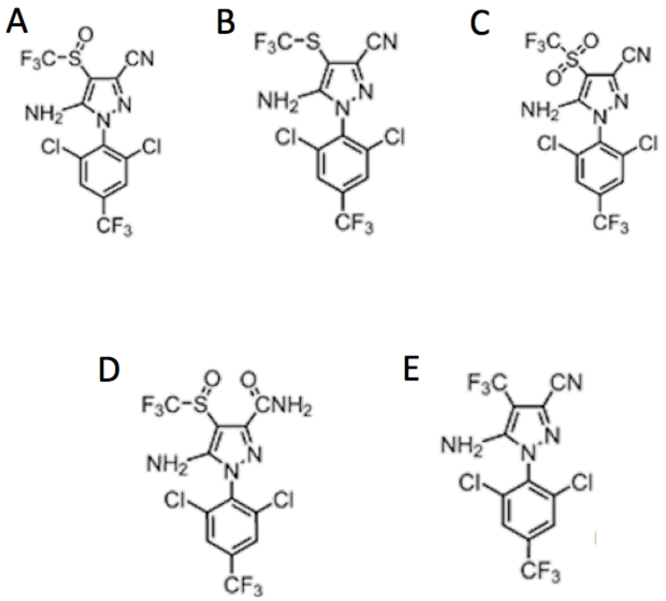

Supplementary Figure S2. Chemical structures of the parental pesticide, fipronil (A), and its transformation products fipronil sulfide (B), fipronil sulfone (C), fipronil amide (D), and fipronil-desulfinyl (E).

References

1. USEPA, Appendix B to Part 136 - Definition and procedure for the determination of the method detection limit-revision 1.11. In 2011.
